# 

**Published:** 1886-03

**Authors:** 


*Entered at the Post Office, at Austin, Texas, as Second-class Matter.*

VOL. I.] **MARCH, 1886.** [No. 9.

### DANIEL'S MEDICAL JOURNAL

SIT LUX

*Subscription: $2.00 a Year in Advance; - - - Single Copies, 25 Cents.*

F. E. DANIEL, M. D., Editor and Publisher, AUSTIN, TEXAS.

NORTHERN OFFICE, 1217 FILBERT STREET, PHILADELPHIA.

*EUGENE VON BOECKMANN, PRINTER, AUSTIN, TEXAS.*

